# Modulation of the northward penetration of Antarctica intermediate waters into the eastern equatorial Indian Ocean under glacial and interglacial conditions

**DOI:** 10.1038/s41598-024-57411-5

**Published:** 2024-03-20

**Authors:** Sandrine Le Houedec, Maxime Tremblin, Amaury Champion, Elias Samankassou

**Affiliations:** Department of Earth Sciences, University of Geneva, Rue des Maraîchers 13, 1205 Geneva, Switzerland

**Keywords:** Palaeoceanography, Palaeoclimate, Marine chemistry

## Abstract

The Indo-Pacific warm pool is the warmest and most dynamic ocean–atmosphere-climate system on Earth and was subject to significant climate changes during the Pleistocene glacial-interglacial transitions. This has been shown to significantly affected the strength of surface waters that redistribute heat from the tropics to the southern part of the Indian Ocean. Here we investigate the response of the oceanic circulation at intermediate depth (1200 m) of the eastern equatorial Indian Ocean (EEIO) with neodymium (Nd) isotopes in the context of the climatic oscillation of the last 500 ka. The most striking feature of our new dataset is the seesaw Nd record that mimics glacial-interglacial cycles. While the interglacial periods are characterized by a higher contribution of the less radiogenic neodymium (~ − 7ε_Nd_) Antarctic Intermediate Water (AAIW), the glacial periods are characterized by more radiogenic water mass of Pacific origin (~ − 5ε_Nd_). To explain the increase in the ε_Nd_ signature toward a more radiogenic signature as the Indo-Pacific connection is reduced under the low sea level of the glacial periods, we show that under global cooling, the AAIW advances northward into the tropics, which is a consequence of the general slowdown of the thermohaline circulation. Therefore, oceanic mixing at intermediate depth in the eastern tropical Indian intermediate water is modulated by the production rate of the AAIW in the Southern Ocean. Our study provides new evidence for the role that changes in the deep oceanic conditions play in amplifying externally forced climate changes that ultimately lead to drier/moister atmospheric conditions and weaker/stronger monsoons during glacial/interglacial periods over eastern tropical Indian Ocean.

## Introduction

The East Equatorial Indian Ocean (EEIO) represents the western part of the larger Indo-Pacific Warm Pool (IPWP). This equatorial region holds a key position on the globe, characterized by sea surface temperature (SST) consistently above 28 °C^1^ and strong rainfall, and contributes to the northward distribution of heat through high convective clouds^2^. The EEIO plays an active role in regulating global climate change^3,4^ and has been classified as one of the major climate “hotspots” by the 5th and 6th successive IPCC reports^5–7^. Indeed, ocean–atmosphere processes are strongly coupled in this area, and despite relatively steady and warm SST, salinity varies considerably due to seasonal Australian-Indonesian monsoonal activity. Due to the central role of EEIO on the global thermohaline circulation, its property fluctuations (temperature and salinity) play an active role in regulating global oceanic dynamics, global energy transfers, and global and regional climate^2,8^. The intermediate water masses originating from the southern hemisphere are an essential component of the thermohaline circulation, and there is growing evidence that these water masses play a significant role in the climate regulation of the Indian Ocean^9–11^. As an example, the Antarctic Intermediate Water (AAIW) is thought to transmit climate anomalies (i.e. through thermohaline properties, nutrients and inorganic carbon) from the Southern Ocean to the low latitude thermocline via the so-called “oceanic tunnel”^12–14^. This term describes the intermediate water level pathway where southern-sourced waters flow towards the tropics and mitigate the tropical SST^15–18^. Collectively these studies support the idea that the vigor of the AAIW is one of the control parameters of the EEIO climate variability, highlighting a direct and significant link between oceanic and atmospheric processes in this crucial area of the Earth’s climate system.

It is well known that the EEIO experienced significant climate changes as response to the global Pleistocene glacial/interglacial cycles^19–21^, with SST oscillating between 26 and 22 °C^22,23^. However, past changes in the intermediate circulation are far less well‐documented than those of surface and deep water^24–27^. Moreover, most of the studies of the intermediate water circulation focus on the last glaciation and are mainly derived from the Atlantic Ocean^26,28,29^ and the Pacific Ocean^30,31^. The response of the AAIW to climatic change remains still understudied, and while some studies suggest an increased northward penetration of the AAIW in those oceans over the last glacial period^29,31,32^, others based on ɛ_Nd_ and Cd/Ca records did not identify such changes^33,34^. For the Indian Ocean, a few studies provide information on the AAIW dynamics and highlight an increased contribution of this current during the last deglaciation into the northern basins^9–11,27^ but none investigate the AAIW dynamics for the pre-LGM period. In this study we generated a high-resolution Neodymium-based oceanographic record together along with climatic proxies (δ^18^O and Mg/Ca) to reconstruct the coupled ocean–atmosphere response of the EEIO to the 100-ka eccentricity climatic cycles of the last 500 ka in the EEIO.

### Oceanographical settings

**Figure 1:**
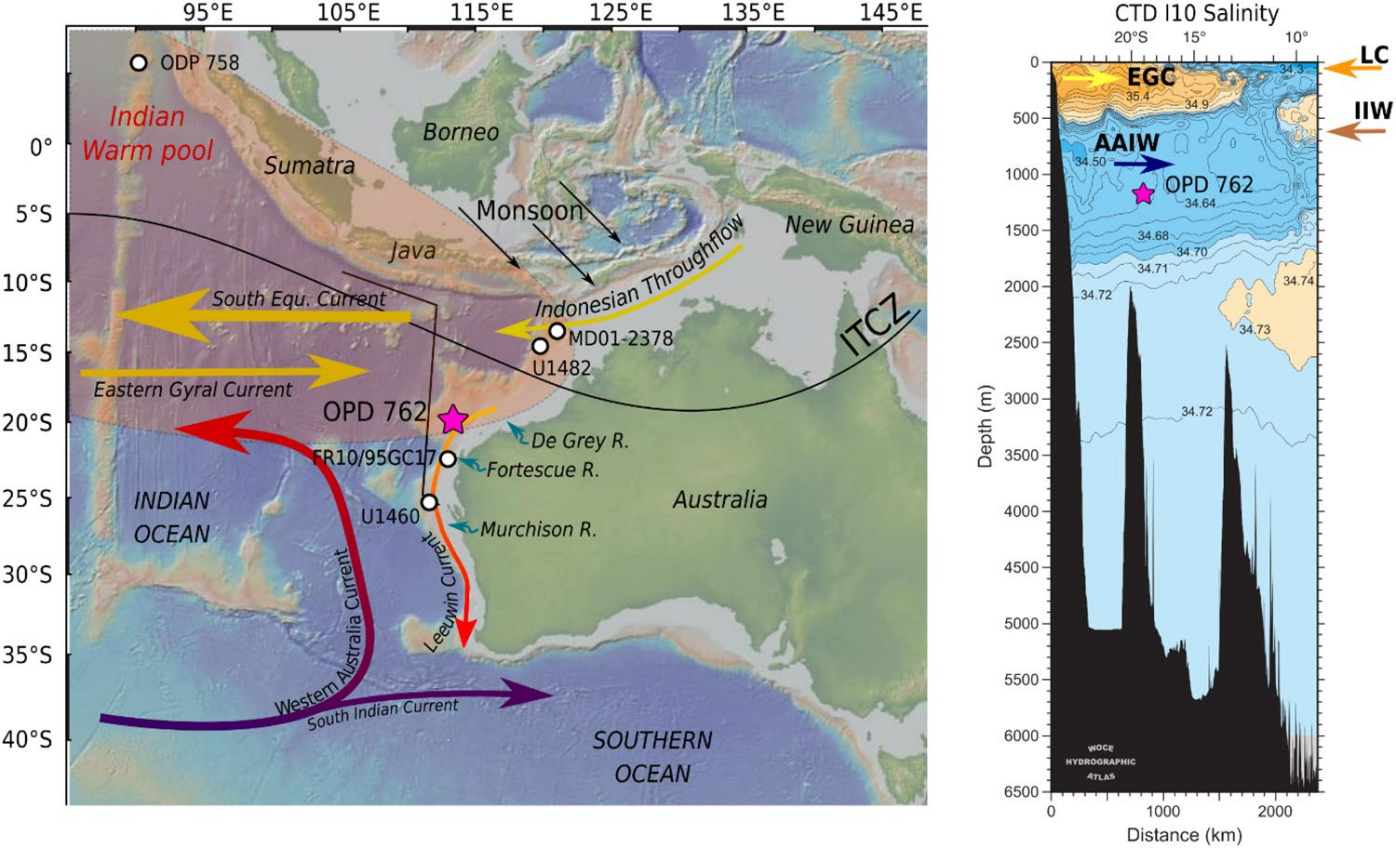
Main environmental and oceanographic features of the Equatorial East Indian Ocean. Left panel: Map that illustrates the surface currents in the EEIO. The pink star shows the current position of the study site ODP 762. Blue arrows show the outlet of the river near to the site ODP 762. The map was generated using map-based application GeoMapApp (http://www.geomapapp.org). Right panel: Salinity profile along CTD salinity profile I10 110° E (WOCE hydrographic surveys). The main water masses are marked: EGC, Eastern Gyral current; LC, Leeuwin current; IIW, Intermediate Indonesian Water; AAIW, Antarctic Intermediate Water. The Intertropical Convergence Zone (ITCZ) position corresponds to its Austral summer position (December -January).

The ODP Site 762 (Hole B) is located on the Exmouth Plateau in the Western Australian Margin (19°53′ S, 112°15′ E) and was drilled at intermediate ocean depth (water depth = 1360 m). The sedimentary succession is mainly composed of well-preserved carbonated oozes. The ages were calculated using the age‐depth tie points of the stratigraphically integrated age model for the last 6 Ma of the site ODP 762 Hole B^35^. Currently, the main features of the surface circulation affecting the Exmouth plateau are the Indonesian Throughflow (ITF), the West Australian Current (WAC, > 400 m), and the Leeuwin Current (LC, > 200 m) (Fig. 1). The ITF consists of warm and low-salinity waters flowing from the Pacific Ocean into the Indian Ocean through the Indonesian pathway^36^. The ITF surface waters diverge westward into the South Equatorial Current (SEC) and southward along the coast of west Australia, forming the warm and low‐salinity LC^36–38^ (Fig. 1). In addition to the influence of the ITF flow, the strength of the LC is partly controlled by the Australian-Indonesian monsoonal activity^39^. The WAC originates in the southern Indian Ocean^36^ and is a cold water mass that flows northward to join the SEC near the tropics. These surface currents associated with the counter Eastern Gyral Current (EGC) allow the settlement of warm and salty waters that constitute the Indian warm pool (Fig. 1). Associated to these surface currents, two intermediate water currents affect the Exmouth plateau and influence the water column stratification of the study site: the Indonesian Intermediate water (IIW) and the Antarctic Intermediate Water (AAIW). The sill depth between the Pacific and Indian oceans at around 1250 m (Leti Strait^40^), leads to water flow of Pacific origin at an intermediate depth, namely the IIW^41^. The AAIW originates around the Drake passage^42^ and is characterized by a minimum salinity and flows below the subtropical gyre toward the study site (Fig. 1).

## Results and discussion

**Figure 2:**
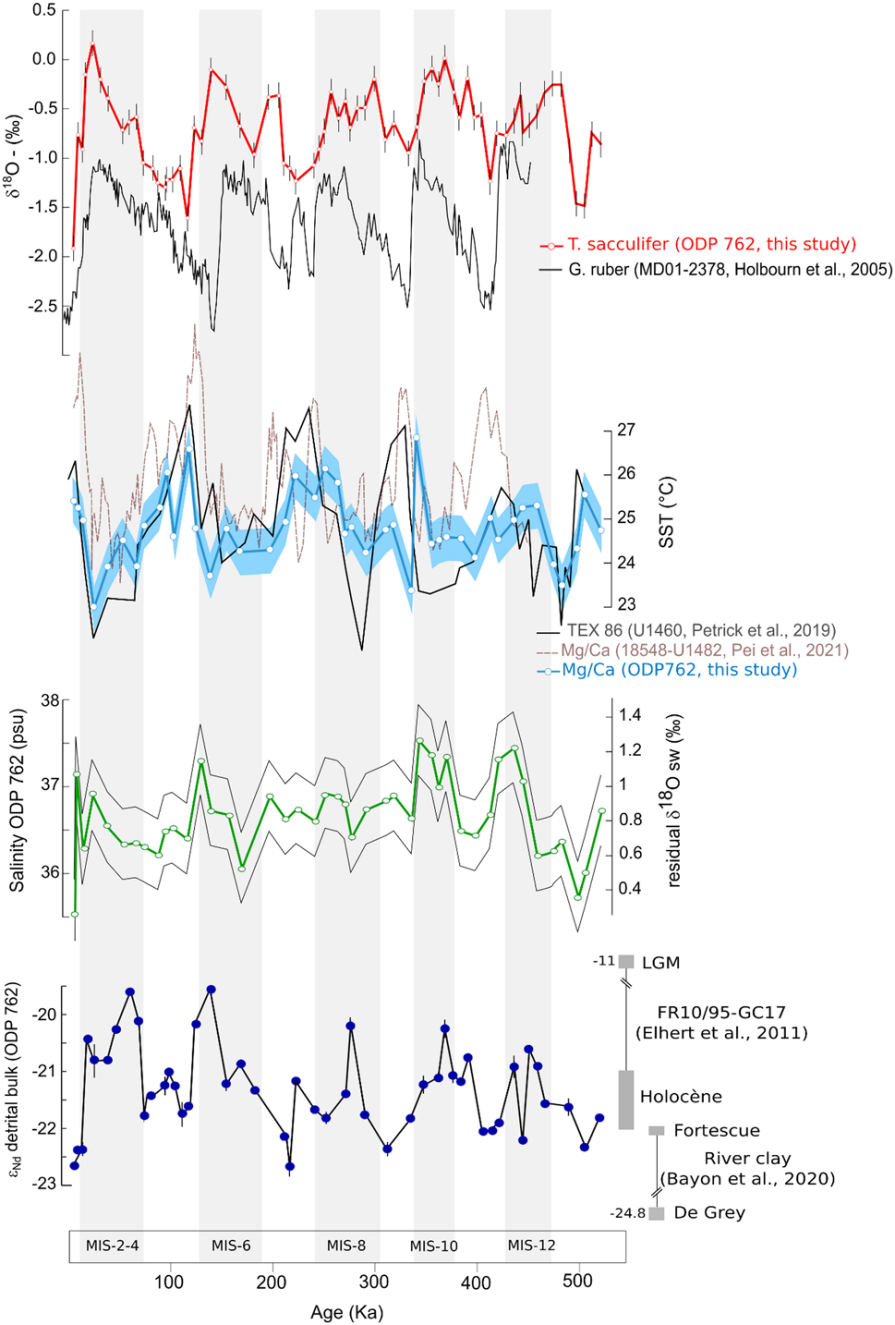
Hydrological and climatic evolution of the East Equatorial Indian Ocean over the last 500 ka. The grey bars highlight the glacial interval. From top to bottom: δ^18^O evolution of *T. sacculifer* (in red) at site ODP 762 (this study) and *G. ruber* (in black) at site MD01-2378^60^, SST evolution of the EEIO during the last 500 ka. TEX_86_ SST derived from the site U1460^23^ (in black), and Mg/Ca temperatures derived from site 18548-U1482^22^ (in red) and from site ODP 762 (in blue, this study), SSS evolution during the last 500 ka calculated from the equation δ^18^O_sw_ = 0.54 × S−18.7^61^, and Nd isotopic composition from terrigenous phases of sites ODP 762 (this study) in the context of the Australian rivers Nd isotopic signatures.

**Figure 3:**
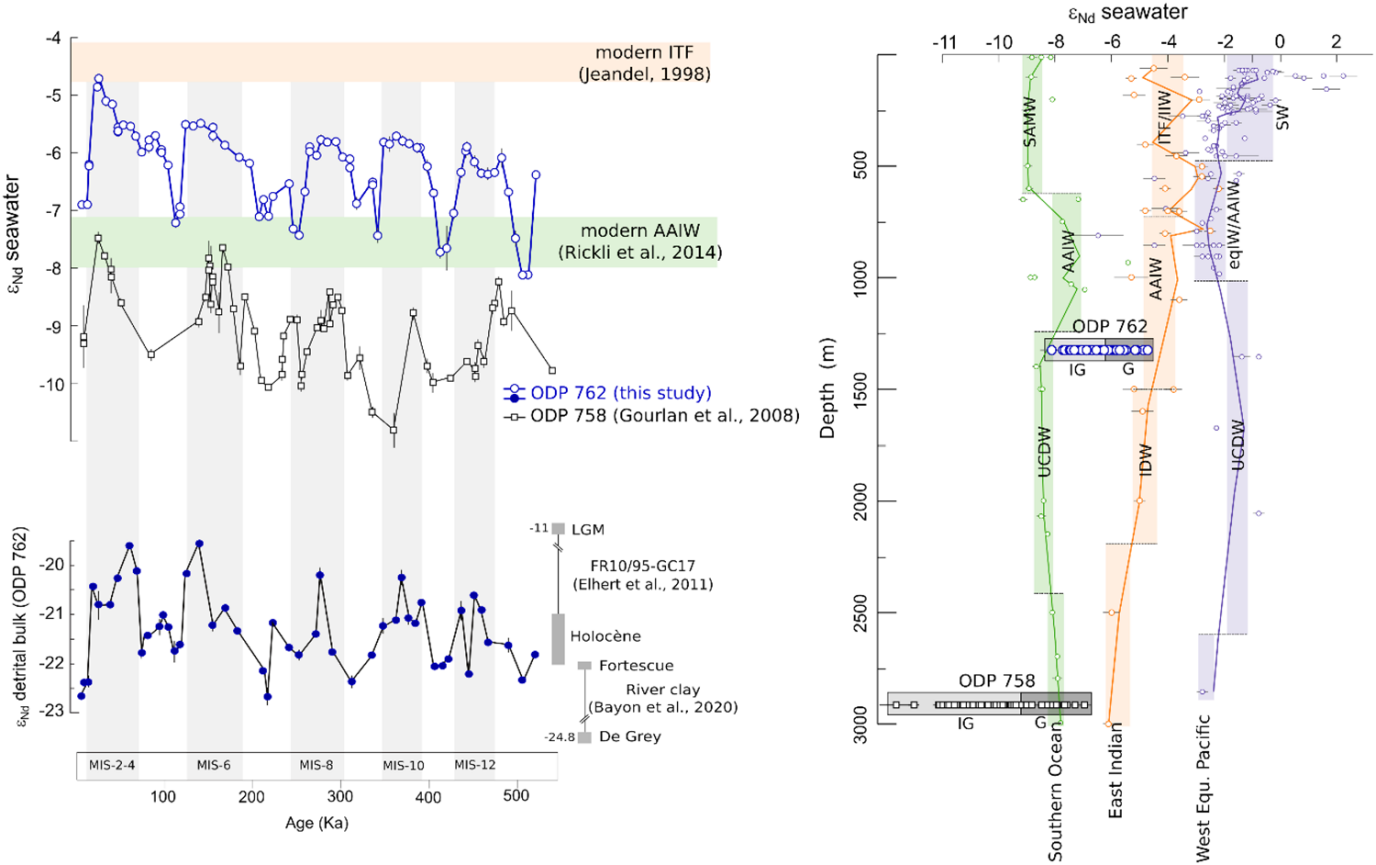
Compilation of regional Neodymium isotopic records. Left panel illustrates the Nd isotopic composition from both carbonate and terrigenous phases of sites ODP 762 (this study) and ODP 758^62^. Right panel shows the Nd isotopic composition of the modern ocean collected from GEOTRACEs database^75^ for the Southern Ocean^70^, East Indian Ocean^76,77^ and West Equatorial Pacific Ocean^78,79^. The main water masses are marked: Subantarctic Mode Water (SAMW), Antarctic Intermediate Water (AAIW), Upper Circumpolar Deep Water (UCDW), Indonesian Throughflow and Intermediate Indonesian Water (ITF/IIW), Indian Deep Water (IDW), Surface Water (SW), Equatorial Intermediate Water (EQIW). Shadow area is the average ε_Nd_ value for each water mass.

Over the last 500 ka, our new multi-proxy data generated at the site ODP 762 follows the global glacial-interglacial (G-IG) climatic cyclicity. The sea surface temperature (SST) oscillates between 27 °C during IG and 23 °C during G (Fig. 2). On average, the SST range of variation is about 2.5 °C between successive G-IG episodes. The sea surface salinity (SSS) follows the G-IG cycles with overall rising SSS values over the glacial and decreasing values over the interglacial episodes and a range of variation at about 2.5 psu (from ~ 23.5 to ~ 26.5 ± 0.7 psu; Fig. 2). The Nd isotope values from the detrital component of the sediment vary from − 23.5 to − 19.5 ε_Nd_ with lower values reached during the IG and the highest values during the G (Fig. 2). The ε_Nd_ values of past seawater inferred range from − 8 to − 5 in a long-term increasing trend from 500 ka to modern time. This long-term trend is featured by embedded variations of 1.5–2 ε_Nd_ units that define the G-IG cycles (Fig. 3).

### Enhanced river activity on the Western Australian margin under interglacial periods

The Western Australian margin is characterized by low salinity waters, which originate from the Indonesian archipelago through the Indonesian Throughflow (ITF) and local precipitation that freshen the surface layer along the pathway of the Leeuwin current^43^ (LC). For instance, the southern position of the Intertropical Convergence Zone (ITCZ) during warm periods results in enhanced monsoonal activity in the area^44–48^. Additionally, sea level fluctuation in response to the G-IG cycles controls the water flux between the Pacific and the Indian Oceans which, in turn, control the strength of the ITF and the LC that carry warm and fresh waters along the western Australia margin^22,43,49^ and even towards the western Indian margin^50^. The migration of salinity fronts is thus influenced by intricate interactions between ITF/LC dynamics and precipitation rates, both of which are modulated from seasonal to orbital scales^22,43,51^.

In this study we used Nd and Sr isotopes to identify the provenance of the terrigenous fractions at Site ODP 762 (Fig. 1, Supplementary material Fig. S1) to monitor local river runoff and infer the monsoonal activity over the last 500 ka. The terrigenous sediment flux is thought to more directly capture the local monsoonal precipitation and runoff signal from the Australian continent^49,52^. Indeed, combinations of Nd, Sr, and Pb isotope ratios on the clay fractions at Site FR10/95‐GC17 (Fig. 1), southeast of Site ODP 762, demonstrated that terrigenous particles in the area are predominantly riverine sources from west Australia during the warm Holocene^49^. In addition, eolian dust from north-western Australia should contribute little to the terrigenous fraction at Site ODP 762, as supported by the estimated terrigenous dust accumulation offshore north-western Australia^53^. We found that Nd isotope values of terrigenous particles during the IG periods are very close to the Nd isotope values of clay minerals from the nearby Fortescue and/or De Grey rivers (Fig. 1) with ɛ_Nd-clay_ = − 21.5 and − 23.4, respectively^54^ indicating the western Australia river’s origin of the terrigenous fractions at Site ODP 762 during the IG periods. The consistency of the Nd isotope values over the IG intervals suggests that the provenance of the terrigenous fractions at site ODP 762 has been relatively stable over interglacial intervals of the past 500 ka.

At site ODP 762, the drop in salinity occurred abruptly at the onset of most of the deglaciation events and is synchronous with a SST warming (Fig. 2, Supplementary material Fig. S2). These environmental changes are consistent with records from the nearby Core SO257-18571, where the drop in salinity and rise in SST at the termination has been attributed to the enhancement of “Ningaloo Niño”—type events^55^ during the *p*CO_2_ peak that follows glacial terminations^22^. These events trigger intense precipitation in the Indonesian region and western Australia^56^ allowing large amounts of warm and fresh water to be transported along the western Australian margin through the LC. In this region, the hydrology cycle is known to follow precession cycle^57,58^ but precipitation rate depend of ITCZ migration and sea level, which is slightly different for each climatic cycles^59^. Therefore, the weaker change in our salinity record for MIS8 might result of a less intense “Ningaloo Nino” type event at that time. In addition, we suggest that during deglaciation, those events of intense precipitation also trigger an increase in the western Australia river’s activity, most likely through flash flood phenomena. The significant input of river water to the Ocean will enhance SSS freshening of the ITF/LC current. This hypothesis is supported by the ε_Nd_ record from the terrigenous particles at site ODP 762, which shows a sharp decrease in the ε_Nd_ values (from 1 to 2 ɛ_Nd_ units) at the glacial termination, confirming a massive release of particles from nearby rivers (Fig. 2). While not ruling out the primary role of the ITF/LC strength on SSS modulation, our data support the hypothesis of a non-negligible SSS control by local river activities. Therefore, combined with the intensification of the ITF under higher sea level stand, the reinforcement of the western Australian rivers’ activity at the deglaciation will exert a positive feedback on the decreasing salinity of the surface waters. We suggest that it is the combination of the two phenomena, ITF strength and river’s activity, which might explain such a large drop of sea surface salinity observed at the climatic transition in the eastern equatorial Indian Ocean.

### Modification of the Eastern Equatorial Indian water column structure over glacial-interglacial cycles

Our study supports an overall colder and saltier surface layer in the EEIO during glacial periods and a warmer and fresher surface layer during interglacial periods. The authigenic ε_Nd_ at site ODP 762 oscillates in response to the G/IG cycles, with the signature driven by radiogenic Pacific water during glacial periods and a signature driven by the less radiogenic Southern Ocean during interglacial periods (Fig. 3, Supplementary material Fig. S2). The ε_Nd_ signature is expected to shift to less radiogenic values during glacial periods due to reduced oceanic connections between the Pacific and the EEIO^23^. Although a tectonic closure of an oceanic passage could potentially increase water flux through a bottleneck effect^62,63^, our isotopic analyses indicate that this is unlikely to occur in the studied area during glacial periods. Indeed, the analyses of Nd isotopes (and Sr isotopes, Supplementary material Fig. S1) in terrigenous particles are too negative (ε_Nd_ = − 20 to − 19, Fig. 3) to support the hypothesis of an increase in particle input from the Indonesian region during these periods (Sumatra/Banda clays^49^ ε_Nd_ = − 13 to − 3). The strength of the northward penetration of the Southern Ocean AAIW into the EEIO in response to global climate resolves this Indo-Pacific ε_Nd_ conundrum. Over the last 40 ka, evidence of northward penetration of the AAIW of Southern Ocean origin was reported for the northern Bay of Bengal^64^ and attributed to episodes of warming during the last deglaciation in Antarctica and the Southern Hemisphere^65^. This mechanism has been linked to the modulation of the sea-ice coverage^66,67^. Indeed, it was modelled that under a warming climate, the AAIW shift poleward together with the surface salinity minimum due to the retreating sea ice^67^. Our new ε_Nd_ results show that similar processes also occur on the eccentricity time scale. Thus, under warmer intervals, the low Antarctic sea-ice coverage enhances the stratification in the Southern Ocean which will promote the production of subsurface and intermediate water masses, including the AAIW and its northward penetration. Consequently, the strong signature of the AAIW from the Southern Ocean, represented by a less radiogenic ε_Nd_ signature, will dominate in the EEIO during these deglaciations. In stark contrast, reduced AAIW during glacial periods causes the influence of less radiogenic AAIW waters in the eastern Indian Ocean to decrease despite the constriction of the Indo-Pacific water flow. Thus, the seesaw trend in ε_Nd_ of the intermediate water is directly derived from the imbalance between AAIW and ITF/IIW in the intermediate oceanic mixing of the EEIO. Superimposed on this seesaw pattern, a long-term increase of the ε_Nd_ value is also observed throughout the last 500 ka. This trend reflects the overall global increase of the seawater ε_Nd_ relative to the present value since the Mid-Pleistocene transition^68^ (MPT).

**Figure 4:**
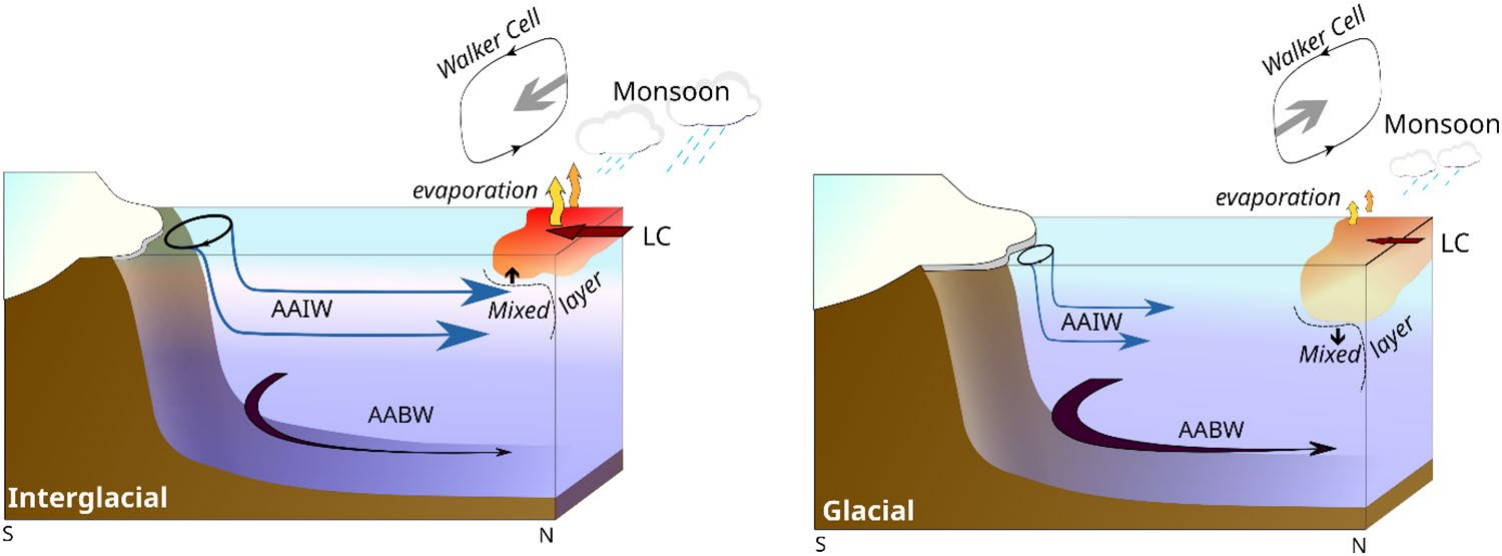
Scenarios of climatic and oceanographic behavior under glacial and interglacial conditions at the EEIO.

To further our understanding of the surface-to-bottom water column structure, we compared our record to the ε_Nd_ signature obtained from the deeper site^62^ (ODP 758, 2925 mbsl), located northwest of our studied site (ninety East Ridge, 5°23′ N, 90°21′ E, Fig. 1). This deep site mimics the G/IG oscillation identified in our study (Fig. 3) showing that the deep current also responds to eccentricity climate cycles. This deep-water Nd record has been interpreted to reflect a weakening and/or less radiogenic Antarctic Bottom Water (AABW) during interglacials^11,69^, which is today^34,70^ between − 6.0 and − 7.8 ε_Nd_, It was shown that reduce AABW under warming climate result of the melting of the sea ice cover off Antarctica in the Southern Oceans decreasing the surface density flux and thus causing change in the deep circulation^71,72^. This in turn implies that the strength of the North Atlantic Deep Water was stronger during the interglacial periods^71,73,74^. Collectively, these results reveal that the vertical structure of the water column has fundamentally changed during G-IG cycles in the EEIO, with a strong AAIW/weak AABW northward flux during interglacial, and weak AAIW/strong AABW northward flux during glacial (Fig. 4).

### AAIW as a feedback control parameter on the regional climate

During glacial intervals, our results support the hypothesis of a drastically reduced production of AAIW in the Southern Ocean, which, in turn, hinders its penetration northwards into the tropical Indian Ocean. Moreover, we propose that the AAIW layer in the EEIO will undergoes significant thinning during glacial periods due to additional factors: (1) the deepening of the surface layer caused by increased density, resulting from colder and saltier surface water, and (2) the expansion of AABW volume due to a high production rate. Conversely, during interglacial periods, a shallower surface layer and weaker AABW will allow the AAIW to easily penetrate northward, resulting in a strong imprint of the AAIW into the Indian Ocean (Fig. 4). Today, the eastern tropical Indian Ocean experiences a strong upwelling of cool, nutrient-rich waters from deeper layers during the summer monsoon season^80,81^. Indeed, a shallower mixed layer allows more upwelling, which in turn intensifies the monsoon by enhancing the sea-air exchange. On a longer time scale, it has also been shown that the mixed layer was shallower along the Sumatra western coastline during the Holocene than during the Last Glacial Maximum^16^. Therefore, we suggest that under warming conditions, the strengthening of northwards flow of the AAIW into the EEIO will shallow the mixed layer depths, amplifying the ability of the surface layer to exchange heat and moisture with the atmosphere and, ultimately enhancing the activity of the Australian-Indonesian monsoon (Fig. 4). Conversely, under glacial conditions, the weak penetration of the AAIW into the EEIO, combined with a saltier surface layer, will deepen the mixed layer and reduce the efficiency of the ocean–atmosphere exchanges. In this scenario, the modulation of the AAIW flux penetrating the EEIO is a non-negligible parameter amplifying externally forced climate changes. In this context, exploring the behavior of the global intermediate oceanic layer during the Pleistocene climatic oscillation seems essential to fully understand the ocean–atmosphere coupling from which the specific spatial expression of global climatic changes is derived. Finally, our new results highlight the need to integrate an intermediate oceanic layer into numerical simulation in order to complete our understanding of the response of the monsoon activity in the context of ongoing global climate change.

## Methods

### Nd measurements

The Nd isotopic data of ancient seawater was obtained from leachates of carbonate bulk samples (400 mg). In the studied area, it was shown that the acetic acid dissolution (1.6 N) of bulk sediment gave the same results as measurements done on isolated foraminifera due to the high CaCO_3_ content (> 85%) of the samples^62,63,82^. Neodymium has been separated using two ion chromatography columns^83^ using Eichrom TRU-Spec™ resin and the Eichrom LnSpec™ resin. The entire procedure Nd blanks were monitored to be smaller than 2 pg and thus is negligible compared to the Nd content of samples (around 1000 ppm). Nd isotopic ratios were measured on a Neptune MC-ICP-MS (Thermo Finnigan). The Nd isotopic results are expressed in ε_Nd_ as:

$$\varepsilon_{{{\text{Nd}}}} = \, \left[ {\left( {^{{{143}}} {\text{Nd}}/^{{{144}}} {\text{Nd}}_{{{\text{sample}}}} } \right)/\left( {^{{{143}}} {\text{Nd}}/^{{{144}}} {\text{Nd}}_{{{\text{CHUR}}}} } \right) \, - { 1}} \right] \, *{ 1}0000$$

with ^143^Nd/^144^Nd_CHUR_^84^ = 0.512638.

The average external reproducibility of the Nd isotopic measurements was monitored by multiple analyses of NIST 3135 A standard (^143^Nd/^144^Nd of 0.511411; 2σ = 7 × 10^−6^; n = 206). The accuracy on ε_Nd_ is calculated to ± 0.2 (2σ). The residual fraction was leached and rinsed by an alternation of three distilled-water rinses and two HBr (1 N) leaches to provide detrital fractions cleaned from residual carbonate and/or Fe–Mn oxides^85^. Then, the fractions were first dissolved by a mixture of distilled HNO_3_ (16 N) and HF (27 N, 0.5 mL for 50 mg) on a hot plate for 24 h, then evaporated. The residual materials were dissolved again using HNO_3_ (16 N), and purified H_3_BO_3_ (9 N, for Sr) was added to remove CaF_2_ crystals^85^. Nd and Sr isotopes of the detrital component were purified on columns from this final solution^86^.

### Mg/Ca measurements

Between 25 and 30 shells of *Trilobus sacculifer* were handpicked and cleaned following the procedure of Barker et al., 2003. The shells were gently crushed using two clean glass plates to open the chambers, then they were cleaned using successive baths of double-distilled water (DDW), glacial ethanol, and alkali buffered 1% H_2_O_2_ in an ultrasonic bath to remove successively clays and organic matter. A final weak acid leach was done using 0.001 M HNO_3_ to remove any adsorbed contaminants from the test fragments. The Mg/Ca ratio was measured on an iCAP 6000 ICP–OES at the University of Geneva. Both Mg and Ca were radially measured on 280.270 and 317.933 nm, respectively. Each single Mg/Ca ratio is the average of three measurements from which the analytical errors are deduced. Mg/Ca ratios were normalized to the JCP-1 certified material^87^. The external reproducibility was obtained using replicate samples with an average standard deviation of 0.024 mmol/mol and measurements of multiples. JCP-1 standard solutions at concentrations similar to those of the measured samples give Mg/Ca = 4.199 ± 0.02 mmol/mol (2σ, n = 42). The sea surface temperature (SST) was obtained from *T. sacculifer* Mg/Ca ratios using MgCarb software^88^, currently recognized to be the best method to obtain accurate temperature from Mg/Ca measurements of foraminiferal shells^89^.

### δ^18^O measurements

δ^18^O was analyzed on a mix of 5–7 shells of *T. sacculifer* (without the last chamber). Shells were handpicked in the size fraction of 300–400 μm and cleaned in DDW in an ultrasonic bath to remove nannofossil oozes and clays. The δ^18^O values were determined from the CO_2_ extracted at 90 °C on an ISOCARB™ device on-line with a VG-PRISM™ mass spectrometer at GEOTOP laboratory at the University of Montréal (UQAM). The values are expressed against the V-PDB standard. The overall analytical reproducibility, as calculated from replicate measurements on the in-house reference standard carbonate material (δ^18^O = − 1.30‰) normalized to the VSMOW-SLAP with a reproducibility of ± 0.05‰ (± 1 s). Sea surface salinity (SSS) was derived from *T. sacculifer* δ^18^O measurements ratios. The δ^18^O of seawater was calculated from δ^18^O of foraminiferal calcite and Mg/Ca-based temperature estimates of *T. sacculifer* based on the equation established for this species^90^. Then, we corrected this calculated δ^18^O seawater value from the global seawater value^91^ to account for the ice volume with a cumulative error estimation of *δ*^18^O_sw_ of ∼ 0.25‰. The residual δ^18^O signal was used to calculate the salinity using the *δ*^18^O_sw_–salinity relationship^92^.

### Supplementary Information

Supplementary Figures.

## Data Availability

The dataset generated for the current study is available in the PANGEA repository: https://doi.org/10.1594/PANGAEA.96092.
