# Supplementary material for: Modulation of the northward penetration of Antarctica intermediate waters into the eastern equatorial Indian Ocean under glacial and interglacial conditions
Source: Sci Rep. 2024 Mar 20;14:6673. doi: 10.1038/s41598-024-57411-5 (PMC11349886; doi:10.1038/s41598-024-57411-5)

Supplementary information for the manuscript titled:

Modulation of the northward penetration of Antarctica intermediate waters into the eastern equatorial Indian Ocean under glacial and interglacial conditions

Sandrine Le Houedec[1]*, Maxime Tremblin[1], Amaury Champion[1], Elias Samankassou[1]

[1] *University of Geneva, Department of Earth Sciences, Rue des Maraîchers 13, CH-1205 Genève, Switzerland.*

(sandrine.lehouedec@unige.ch, maxime.tremblin@unige.ch, Amaury.Champion@crealp.vs.ch, elias.samankassou@unige.ch)

*corresponding author

Figure S1: Sr and Nd isotope cross plot from terrigenous fractions of ODP 762.

The top panel illustrates a two-end-member relationship between the glacial and interglacial time periods. The bottom panel, redrawn after Ehlert et al., 2011, replace the Nd-Sr cross values from ODP 762 in the general context. The signal obtained at the site ODP 762 shows that terrigenous particles originally belonged to West Australian rocks.

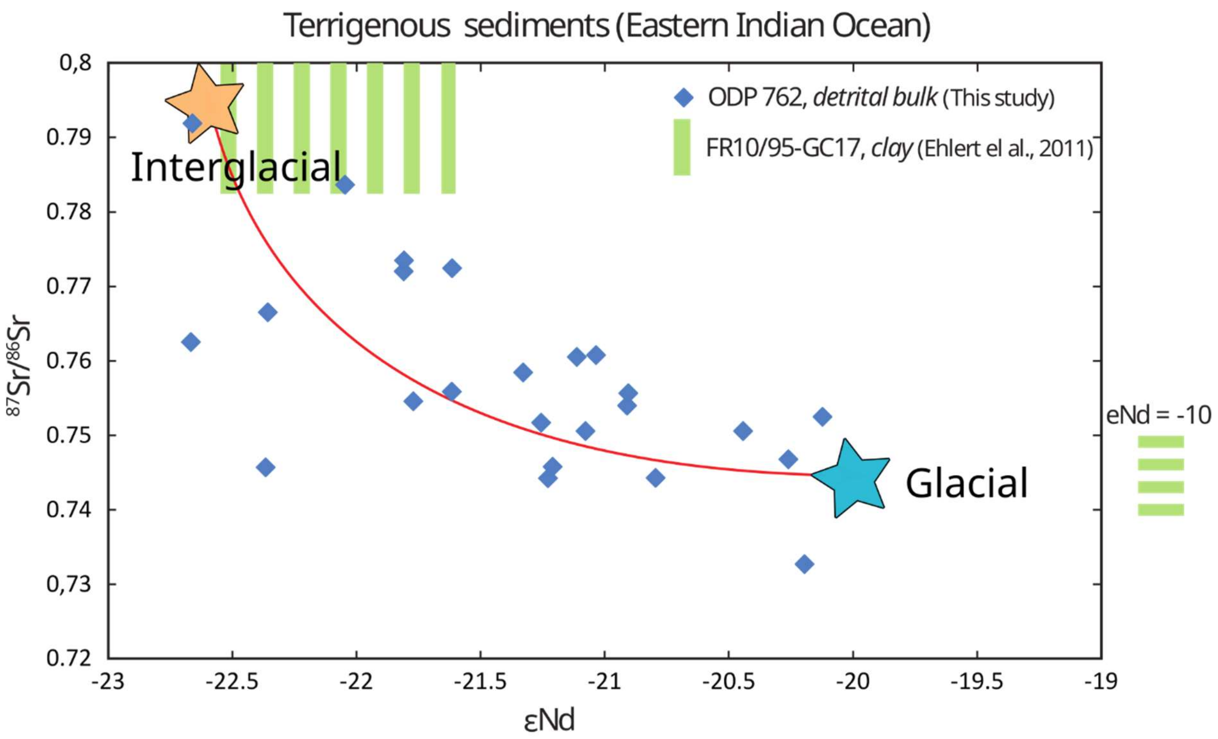

Terrigenous sediments (Eastern Indian Ocean)
ODP 762, detrital bulk (This study)
FR10/95-GC17, clay (Ehlert el al., 2011)
Interglacial
Glacial
eNd = -10
87Sr/86Sr
εNd
0,8
0.79
0.78
0.77
0.76
0.75
0.74
0,73
0.72
-23
-22.5
-22
-21.5
-21
-20.5
-20
-19.5
-19

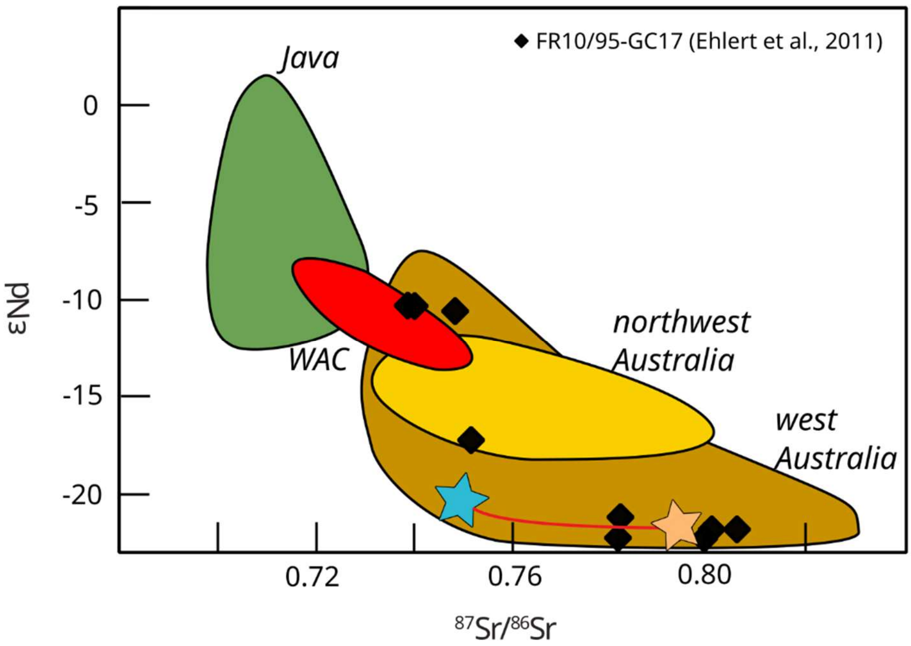

FR10/95-GC17 (Ehlert et al., 2011)
Java
WAC
northwest Australia
west Australia
εNd
87Sr/86Sr
0
-5
-10
-15
-20
0.72
0.76
0.80

Figure S2: Salinity and Temperature histogram plot calculated from Mg/Ca and $\delta^{18}O$ of T. *sacculifer* of ODP 762.

The histogram plots illustrate how salinity and temperature data are distributed during glacial and interglacial stages in terms of frequency. The line on the graph represents the probability of the data distribution (Kernel density) calculated using PAST software. The results indicate a notable trend in the data distribution during glacial episodes, with a significant shift towards higher salinities and relatively lower temperatures. The difference in the distribution of temperatures between glacial and interglacial is less significant due to still relatively low temperature at the beginning of the interglacial (i.e., the termination events.)

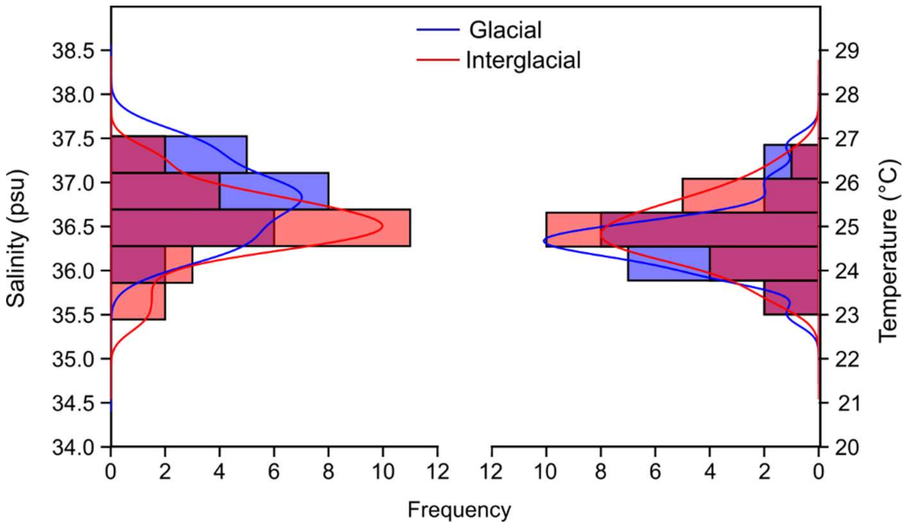

Figure S3: Comparison of sea surface salinity estimation between the eastern and western Indian Ocean inferred from $\delta^{18}O_{sw}$ sea level corrected.

The corrected $\delta^{18}O_{sw\text{-}ivc}$ represents the $\delta^{18}O_{sw}$ value adjusted for the ice volume variation at IODP 1476 in the western Indian Ocean, as reported by Nuber et al., 2023. This corrected value is then compared with our residual $\delta^{18}O_{sw}$, which is calculated as the disparity between the $\delta^{18}O_{sw}$ derived from our data and the global $\delta^{18}O$ seawater value provided by De Boer et al., (2014). The comparison of these datasets indicates that the sea surface salinity change over the last 500 Ka is comparable in both the eastern and western tropical Indian Ocean. Thus, the Indonesian Throughflow (ITF) current connect the two sides of the tropical Indian Ocean and transmits the salinity anomaly from the east to the west.

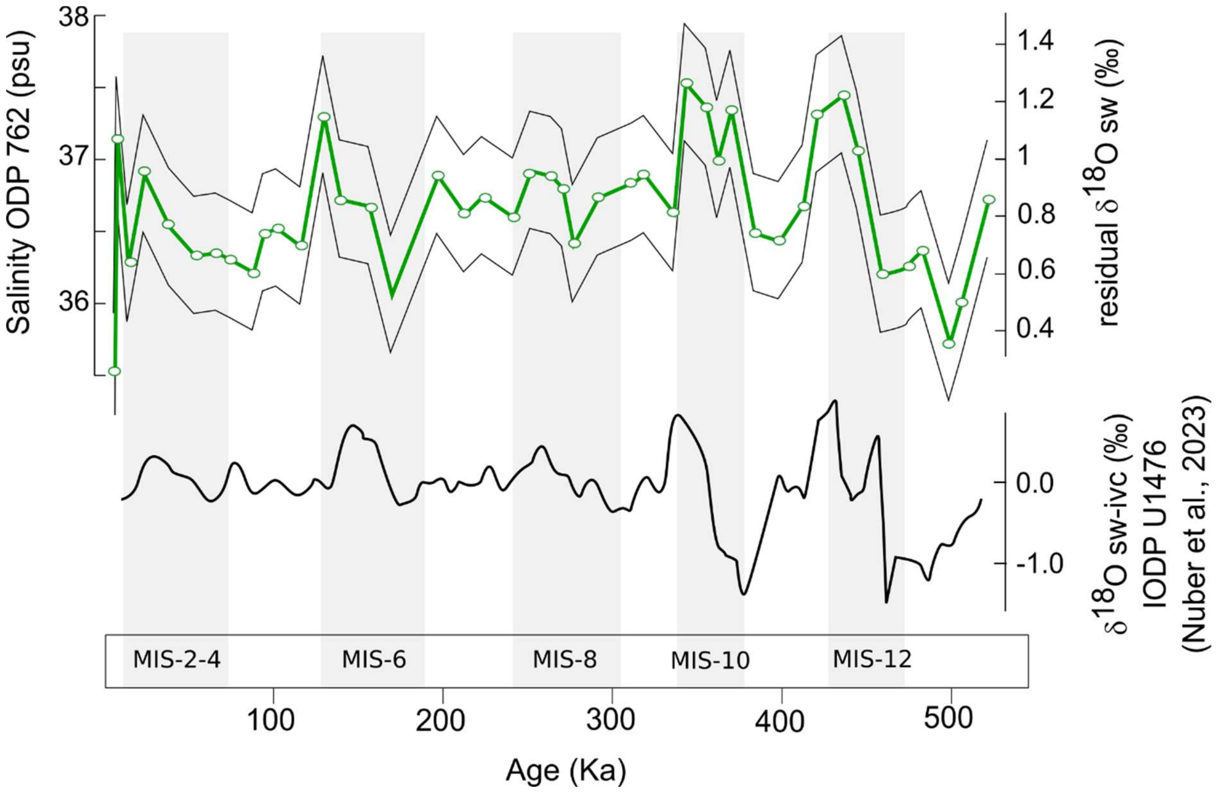

Supplement: Supplementary file 1 — Supplementary Figures. [file 41598_2024_57411_MOESM1_ESM.pdf]
